# Emerging Role of Diet and Microbiota Interactions in Neuroinflammation

**DOI:** 10.3389/fimmu.2018.02067

**Published:** 2018-09-11

**Authors:** Mathangi Janakiraman, Gurumoorthy Krishnamoorthy

**Affiliations:** Research Group Neuroinflammation and Mucosal Immunology, Max Planck Institute of Biochemistry, Martinsried, Germany

**Keywords:** microbiome, EAE, MS, neuroinflammation, diet

## Abstract

Commensal gut microbiota exerts multifarious effects on intestinal and extra-intestinal immune homeostasis. A disruption in the microbial composition of the gut has been associated with many neurological disorders with inflammatory components. Here we review known associations between gut microbiota and neurological disorders. Further we highlight the emerging role of diet and microbiota interrelationship in regulating neuroinflammation.

## Introduction

Inflammation in the nervous system, termed neuroinflammation, is now recognized as a cardinal feature of many neurological disorders. In central nervous system (CNS) autoimmune diseases such as multiple sclerosis, there is a conspicuous infiltration of immune cells from the periphery into the CNS leading to demyelination and neuronal damage (1). However, there is also an elevated expression of inflammatory mediators or modest infiltration of immune cells in neurodegenerative disorders (Parkinson's disease and Alzheimer's disease), acute ischemia, traumatic brain injury, mood disorders, and autism spectrum disorders (2). If uncontrolled, chronic inflammation may have undesirable consequences for healthy status of the nervous system.

Over the years, our understanding of the initiation and regulation of neuroinflammatory processes has been revised. In particular, bidirectional communication between the immune system and the nervous system has been given greater emphasis. Evidence has been mounting in recent years supporting the substantial contribution of several environmental factors including diet and the gut microbiota besides genetic factors in neuroinflammatory diseases. Once thought to be important primarily for conferring nutritional benefits, the gut microbiota has now been implicated in various physiological and pathophysiological responses through direct and indirect interactions with the host (3). Recent advances suggest that the gut-brain axis, the bidirectional communication between the gut commensals and the CNS, involves immune mechanisms, the vagus nerve, and neurometabolite production. In this mini-review, we describe recent studies that investigate the diet—gut microbiota interrelationship in regulating inflammatory processes in neurological diseases.

## Microbiota in neuroinflammation—evidence from experimental models and humans

### Multiple sclerosis (MS) and neuromyelitis optica (NMO)

MS and NMO are the chronic inflammatory demyelinating disorders of the central nervous system that involve the brain, optic nerve and spinal cord with distinct immunological and pathological features. Compelling evidence for the role of the gut microbiota in MS has largely emerged from animal models. Early observations in a spontaneous experimental autoimmune encephalomyelitis (EAE) animal model—a mouse model for MS—suggested a microbial role in initiation of neuroinflammatory disease processes. Goverman et al. showed that transgenic mice harboring high frequencies of myelin basic protein (MBP)-specific T cells in their immune repertoire showed higher spontaneous EAE incidence in conventional dirty facilities compared to clean housing conditions (4). Empirical evidence from many labs also suggested that severity and incidence of actively induced EAE differs depending on the housing conditions. However, the nature of the “microbial” factors remained unclear.

In 2011, using a transgenic mouse model for relapsing-remitting MS (RRMS) under germ free (GF) conditions i.e., mice devoid of microorganisms, a clear role for the gut microbiota in triggering CNS inflammation was established. The relapsing remitting (RR)-MS model—a T cell receptor transgenic mouse strain that is prone to develop spontaneous EAE under specific pathogen free (SPF) housing conditions, remained largely protected from EAE development under GF conditions (5). Similar disease protection was also noted in an actively induced EAE mouse model where GF mice immunized with myelin antigen showed reduced disease severity compared to mice with intact microbiota (6). Further, oral antibiotic treatments, which affect gut microbiota composition, also resulted in disease amelioration in active EAE models with a reduction in pro-inflammatory cytokines and increase in anti-inflammatory cytokines (7, 8). Complementary to these findings, probiotic bacterial strains and commensal isolates were also shown to modulate EAE in active EAE models (9–12).

Inspired by these experimental studies in rodents, several groups have examined a role for the gut microbiota in adult and pediatric MS patients (13–18). These studies have compared the microbiota of MS patients and healthy controls using 16S rRNA sequencing. The results from these studies have shown that there are no dramatic changes in the composition of the gut microbiota between MS patients and healthy controls. Nevertheless, subtle dysbiosis has consistently been observed across these studies. A Japanese study with 20 MS patients noted a depletion of *Clostridia* and *Bacteroidetes* species (13). Another study from USA with 60 MS patients found an increase of *Methanobrevibacter* and *Akkermansia* and decrease of *Butyricimonas* in MS affected individuals (15). Consistent with the increased *Methanobrevibacter*, they found elevated levels of methane in the breath of MS patients (15). Further analysis showed that MS patients on disease-modifying treatment had increased *Prevotella* and *Sutterella* and decreased *Sarcina*, compared with untreated patients (15). A study with 31 MS patients reported increased abundance of the genera *Psuedomonas, Mycoplana, Haemophilus, Blautia*, and *Dorea* with a decrease in *Parabacteroides, Adlercreutzia*, and *Prevotella* genera in MS patients (14). In case of pediatric MS patients, a small longitudinal study found that absence of *Fusobacteria* was associated with increased risk of an earlier relapse (16).

While all the above mentioned studies observed subtle dysbiosis of the microbiota in MS patients, they do not provide any evidence for a role in disease pathogenesis. Two recent studies used human fecal microbiota transplantation approach and showed that MS patient's microbiota contribute to neuroinflammatory disease pathogenesis (17, 18). Berer et al. assessed the microbiota composition in 34 monozygotic twin pairs who are discordant for MS and Cekanaviciute et al. investigated 71 MS patients and healthy controls. In line with other studies, neither group found major shifts in the bacterial community profiles but both groups found differences in the abundance of *Akkermansia*. Most importantly, transplantation of gut microbiota from the twin pairs into GF spontaneous RR-EAE mice resulted in increased EAE incidence in recipients of microbiota from MS-affected twins compared to their healthy counterparts (17). Similarly, microbiota transplants into GF C57BL/6 mice and subsequent EAE induction with MOG peptide immunization resulted in more severe EAE in recipients from MS patients than healthy controls (18). In both cases, IL-10 expression by T cells was found to be decreased in mice receiving MS patient's microbiota (17, 18).

A potential role for the gut microbiota in neuromyelitis optica (NMO) spectrum disorders stemmed from observations that antibodies against intestinal antigens have been found in NMO patients (19). One study found that peripheral blood T cells from NMO patients are cross-reactive with both aquaporin-4 and an adenosine triphosphate-binding cassette transporter from a gut microbe *Clostridium perfringens* (20). More direct evidence came from sequencing analyses which showed the differences in gut microbiota community composition between NMO patients and healthy controls including higher levels of *C. perfringens* in the NMO patients (21).

### Ischemic stroke

Acute brain ischemia induces a local neuroinflammatory reaction and also alters peripheral immune responses. Previous research shows evidence of increased susceptibility to stroke among Crohn's disease patients (22). Conversely, stroke has been shown to cause microbial dysbiosis, which in turn worsens the outcome due to alterations in post stroke immune response. The deteriorated outcome is attributed to increased immune activation in the intestine, with subsequent migration of monocytes and polarized T_H_1 and T_H_17 cells to the CNS. Clinching the effect of microbial dysbiosis in this process was that GF mice receiving microbiota from filamentous middle cerebral artery occlusion (fMCAO) operated mice showed larger infarcts post middle cerebral artery occlusion (MCAO) induction in addition to higher levels of pro-inflammatory cytokines IFNγ and IL-17. Moreover, fecal microbial transplantation restoring a healthy microbiota was neuroprotective (23).

Setting forth a role for microbiota in stroke, dysbiosis induced through administration of broad spectrum antibiotics before stroke induction reduced infarct volume by 60%. The dysbiotic microbiota had expansions in *Proteobacteria* and decrease in *Firmicutes* and *Bacteroides* (24). Neuroprotection due to this dysbiosis was accompanied by increased Treg cells and reduced IL-17^+^ TCRγδ cells (24). Relative frequencies of bacterial families like *Verrucomicrobiaceae, Prevotellaceae*, and *Clostridiaceae* were identified as good predictors of infarct volume (24), implying likely therapeutic possibilities. In contrast, through treatment with different antibiotic regimens, Winek et al. showed that dysbiosis decreased survival of mice after MCAO, implying differential effects of different microbial species on brain injury and recovery (25). Yet another study showed in addition to marked changes in the gut microbiota after stroke, systemic dissemination of some microbes from the gut into the lung, liver, spleen, and broncho-alveolar lavage fluid (26, 27).

### Parkinson's disease

Parkinson's disease (PD), the second most common age-associated neurodegenerative disorder, has pathological hallmarks of chronic neuroinflammation with features of activated glial cells and increased in pro-inflammatory cytokines. The gut microbiota has also been implicated in PD. It is interesting to note that PD is accompanied in many instances by GI symptoms. A study by Devos et al. on PD patients showed pro-inflammatory cytokine signature in the colon corresponding to disease duration. The mRNA levels of TNFα, IL-1β, IFNγ, and IL-6 were increased in colon biopsies from PD patients as compared to healthy controls (28). Another study reported a lower abundance of *Prevotellaceae* in the feces of subjects with PD compared to controls (29). Similarly dysbiosis has also been observed in different PD patient cohorts (30–32). With previous evidence from biopsies of PD patients that α-synuclein aggregation starts in the enteric nervous system and the vagus nerve before progressing to the CNS, Sampson et al. examined the role of the microbiota in motor deficits associated with PD in an α-synuclein overexpressing mouse model. A comparison between GF and SPF mice showed marked decline in motor functions in SPF mice with age, which was delayed in GF mice. Constipation as a measure of GI defects was also pronouncedly more in SPF mice compared to GF mice. GF mice had substantially lesser α-synuclein aggregates in the brain, marked decrease in expression of inflammatory cytokines (TNFα and IL-6) and lesser microglial activation compared to SPF mice. “Humanized” mice with microbiota from PD patients had increased motor deficits compared to mice with microbiota from healthy donors (33).

### Alzheimer's disease

Increasing evidence suggests that Alzheimer's disease (AD) pathogenesis involves strong interactions with innate immunological mechanisms in the brain. In an AD disease mouse model, antibiotic treatment was reported to perturb microbial diversity, alter circulating cytokine and chemokine levels and curtail amyloid plaque deposition (34). One study demonstrated that GF APPPS1-Tg mice exhibit reduced cerebral and serum amyloid-β levels (35). Vogt et al reported a decrease in *Firmicutes* and *Actinobacteria* at the phylum level with increase in abundance of *Bacteroides* in AD patients as opposed to healthy controls. A deeper analysis in AD participants showed reduced abundance at the family level for *Ruminococcaceae, Turicibacteraceae, Peptostreptococcaceae, Clostridiaceae, Bifidobacteriaceae*, and *Mogibacteriaceae*, and increased abundance of *Gemellaceae, Bacteroidaceae, Rikenellaceae* and the genus *Bilophila* in the phylum *Proteobacteria*. Correspondingly, correlations between relative abundance of aforementioned bacteria and CSF biomarkers like Aβ_42_/Aβ_40_ and p-Tau showed consistent trends, with genera identified as more abundant in AD being associated with greater AD pathology, even in non-demented participants (36).

### Autism spectrum disorders (ASD)

Autism is a complex neurodevelopmental condition where neuroinflammation—microglial activation in particular has been observed. In children with regressive-onset ASDs, increased intestinal permeability, diarrhea, and gas retention have been reported alongside too. Consequently, treatment with antibiotics like vancomycin have been seen to transiently improve GI symptoms and cognitive skills in these patients (37). In a model of maternal immune activation (MIA—a mouse model for risk factors in ASD), defects in intestinal integrity and alterations in the commensal microbiota analogous to features in human ASDs were reported. Treatment with *Bacteroides fragilis* was shown to improve gut barrier integrity, restore specific microbiota changes, modulate serum metabolome profile and ameliorate anxiety-like, communicative and sensorimotor behaviors. Interestingly, this improvement was not dependent on Polysaccharide A (previously identified as responsible for immunomodulatory effects mediated by *B. fragilis*). Moreover, no evident colonization by the *B. fragilis* was observed in the GI tract, nor was there change in systemic immunity. Besides, the same behavioral improvements were possible by treatment with *Bacteroides thetaiotaomicron* too but not with *Enterococcus feacalis* (38). These findings not only establish a role for the commensal microbiota in neurodevelopmental disorders, they also highlight the necessity for specificity in microbial treatment to evince therapeutically relevant responses in the host.

## Impact of diet and microbiota interrelationship on neuroinflammatory responses

A plethora of factors can shape the gut microbial community. In particular, diet being a persistent stress can produce wide ranging alterations in microbial diversity and community structure. Indeed, a study with humanized mice showed that the gene profile of the microbial community was significantly altered as early as 1 day after switching diets (39). A similar study in humans showed significant differences in the microbial diversity and community structure as early as 1 day after diet switch. This change seems to be reversible and the microbial community reverted to their original composition within 2 days. In addition to the microbiota changes, the levels of metabolites like SCFAs (short chain fatty acids) and some bile acids were altered in feces from participants with animal-based diets (40).

**Figure 1:**
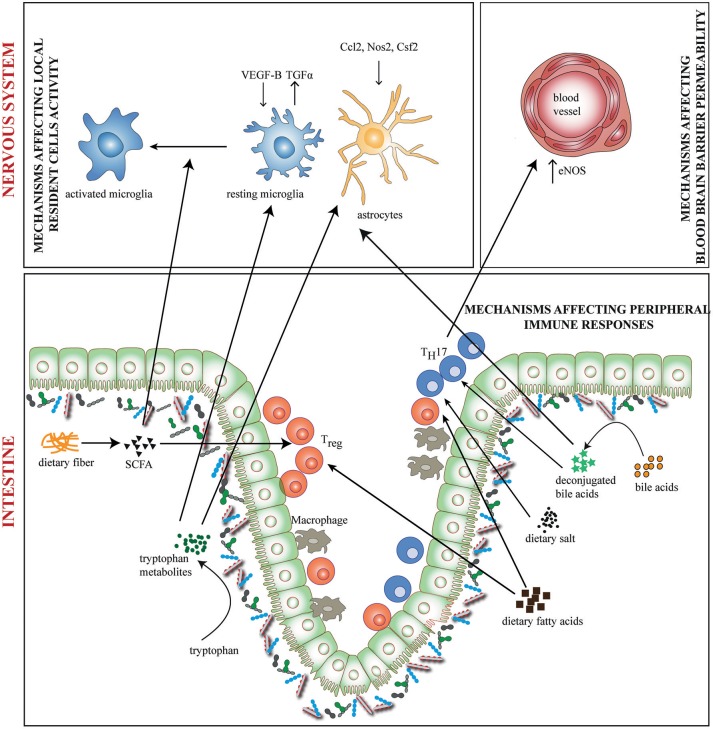
Diet-Microbiome interactions in neuroinflammation. Gut microbiota and dietary metabolites contribute to neuroinflammation through direct and indirect ways. Dietary fiber and tryptophan are modified by the microbiome to generate metabolites such as short chain fatty acids (SCFA) and indole derivatives which affects functional activity of local resident cells—the microglia and astrocytes. Tryptophan metabolites regulate the production of pro-and anti-inflammatory mediators like VEGF-B and TGF-α on microglia and Nos2, Ccl2, and Csf2 on astrocytes. SCFAs regulate the activation of microglia. In addition, bile acids, SCFAs, dietary salt and fatty acids directly induce differentiation of either T_H_17 or T_reg_ cells. This may result in altered activity of inflammation associated molecules. T_H_17 cells upregulate eNOS in CNS endothelia lining the blood vessels, thus altering blood brain barrier permeability. Bile acids may also penetrate the CNS and affect the activity of resident cells.

Diet can have direct or indirect (via the microbiota) effects on neurological disorders. Research in this direction has expanded in recent times producing a multitude of results. The role of diet in neurological disorders has been reviewed elsewhere (41, 42). Here we focus on relevant research that suggests potential involvement of diet and microbiota interactions in neuroinflammation. Gut microbiota and dietary metabolites could affect neuroinflammation in at least 3 ways (a) by affecting functional activity of local resident cells (b) by affecting immune cell entry into the nervous system (c) by affecting peripheral immune responses (Figure 1).

Microglia and astrocytes are the key local resident cells which critically contributes to the inflammatory processes in the CNS. Indeed, it has been shown that the microbiota influences microglial maturation and function (43). GF mice have increased numbers of immature microglia across CNS gray and white matter as evident from longer processes, more branching and elevated expression of colony stimulating factor 1 receptor (CSF1R), F4/80, and CD31 (43). Moreover, microglia from GF mice are functionally impaired and have attenuated immune responses in response to challenge with microbial products (43, 44). The importance of diet-microbiota interrelationship in microglial function has been suggested by the fact that the alterations observed in GF mice were normalized by treatment with microbial fermentation products, SCFA (43). SCFAs may cross the BBB to impact the physiology of microglia or they may have peripheral effects, which indirectly activate and mature microglia. While addressing the impact of SCFA on αSyn-Mediated Neuroinflammation in a PD mouse model, Sampson et al observed an increased microglial activation, αSyn aggregation and motor dysfunction in SCFA treated animals (33). Whether similar effects of SCFA and other dietary metabolites on microglial maturation and function contribute to neuroinflammation in other neurological disorders needs further investigation.

The gut microbiota and microbial metabolites have also been shown to affect astrocyte activity through aryl hydrocarbon receptors (Ahr) (45, 46). Dietary tryptophan is metabolized by gut microbes to produce natural Ahr ligands such as indole-3-aldehyde and indole-3-propionic acid (47). In an EAE model, ampicillin treatment, which depleted certain groups of gut microbiota and reduced the levels of tryptophan metabolite indoxyl-3-sulfate (I3S), worsened EAE disease scores (45). Further, administration of I3S or indole supplemented diet to mice improved EAE symptoms (45). Interestingly, a recent report suggested that metabolites of dietary tryptophan produced by the commensal flora control microglial activation and TGFα and VEGF-B production which are important to promote pro-inflammatory activities in astrocytes (46).

In addition to modulating microglia and astrocytes, gut microbiota, and dietary metabolites may regulate the entry of immune cells into the nervous system by affecting vascular permeability. Using GF mice Braniste et al. reported that gut microbiota affects BBB permeability in both the fetal and adult mouse brain (48). Treatment of GF mice with bacteria that produce dietary fiber fermentation products, SCFA, decreased the permeability of the BBB supporting a role of diet-microbial interactions in BBB permeability (48). High dietary salt (HSD) consumption has also been recently linked to neurovascular dysregulation initiated by gut microbiota induced T_H_17 lymphocytes. IL-17 mediated suppression of endothelial NO via Rho kinase (ROCK)-dependent inhibitory phosphorylation of endothelial NO synthase (eNOS) leads to a cerebral hypoperfusion and alteration in the endothelial regulation of cerebral microcirculation (49). However, the relevance of this altered permeability induced by gut microbiota in neuroinflammatory conditions has not been investigated.

Gut microbiota and dietary molecules are potent regulators of mucosal and systemic immune responses and contribute to the development of neuroinflammation. The most prominent immune response induced by gut microbiota contributing to neuroinflammation are IL-17 producing T_H_17 cells (5, 6). GF mice produce much less T_H_17 cells than conventional mice and mono-association of GF mice with T_H_17 inducing segmented filamentous bacteria (SFB) promoted EAE (6). It is interesting in this context, that high salt exacerbates EAE by inducing differentiation of T_H_17 cells through the NFAT5/SGK1 dependent cascade (50, 51). A role for microbiota and metabolites in HSD mediated effects has been shown in a recent study. In HSD fed animals, they observed a decrease of *Lactobacillus murinus* in the gut and tryptophan metabolites indole-3-lactic acid (ILA) and indole-3-acetic acid (IAA). Mono-association of GF mice with *L. murinus* increased fecal ILA and IAA as well as ameliorated EAE even under high salt diet conditions (52). Apart from tryptophan metabolites produced by the gut microbiota, fiber fermentation products like SCFA, have also been implicated in protection from EAE. Treatment of mice with SCFAs reduces EAE and axonal damage through increased Treg differentiation whereas long chain fatty acids worsen EAE through increased T_H_1 and T_H_17 differentiation (53). In addition to fermentable fibers, non-fermentable fibers such as cellulose which are commonly found in plant based diets, are also shown to affect EAE development by modulating microbiota and immune responses (54). Non-fermentable fiber supplementation led to decreased bile acids levels in the intestine thereby impairing the absorption of dietary long chain fatty acids. Eventually, these resulted in altered immune responses such as decreased pro-inflammatory T_H_1 responses and increased T_H_2 responses, thus favoring suppression of neuroinflammation (54).

Apart from SCFAs, several other microbial metabolites have also been shown to regulate systemic immune responses in neuroinflammatory diseases. One such metabolite, TMAO (Trimethylamine-N-oxide), a product of choline metabolism, has been recently correlated with increased levels of pro-inflammatory monocytes in the blood and bone marrow of stroke patients (55). Another class of important microbiota dependent metabolites are secondary bile acids. Gut microbiota converts primary bile acids synthesized from cholesterol in the liver into secondary bile acids which regulate diverse metabolic pathways in the host through Farsenoid X receptor (FXR), G protein-coupled membrane receptor 5 (TGR5), and the Glucocorticoid receptor (56). Although, the precise contribution of microbiota- derived secondary bile acids to the neuroinflammatory diseases is unknown, bile acid receptor activation is shown to affect neuroinflammatory processes. In an EAE model, 6-ECDCA (Obeticholic acid—a synthetic FXR ligand) and CDCA (chenodeoxycholic acid—a natural FXR ligand) were both seen to ameliorate disease through suppression of pro-inflammatory cytokine production and altering the PD-1 dependent T cell regulation (57). Similarly, activation of FXR through synthetic agonist GW4064 ameliorated EAE by promoting the generation of anti-inflammatory macrophages (58). While it is conceivable that gut microbiota is essential for fiber fermentation to SCFAs, choline, and bile acid metabolism, which species are involved and how they are modulated in natural diet conditions that contributes to neuroinflammation has not been explored.

## Conclusion and future prospects

As reviewed, gut microbiota contributes to the inflammatory processes in neurological disorders. Associating specific microbial communities in patients with neurological disorders has been made easy with improvement in sequencing tools. But the functional relevance of these associations and the mechanisms through which they cause or ameliorate neurological disorders are unexplored. Diet being the primary factor regulating microbial community structure and diversity, further research is needed to find specific dietary factors that shape the commensals and the resulting bioactive metabolites. This will help us in designing new therapeutic interventions through dietary modifications which serve several advantages like being cost effective, simpler to implement and can be personalized therapeutics.

## Author contributions

All authors listed have made a substantial, direct and intellectual contribution to the work, and approved it for publication.

### Conflict of interest statement

The authors declare that the research was conducted in the absence of any commercial or financial relationships that could be construed as a potential conflict of interest.
